# Garden Cities for Consumptives
*The first article appeared on November 1.


**Published:** 1913-11-15

**Authors:** J. E. Esslemont


### GARDEN CITIES FOR CONSUMPTIVES.*

### II.—The Initial Organisation.

By J. E. ESSLEMONT, M.B.

A rough estimate of the cost of an efficient segregation scheme is given by Professor Moore in his masterly essay on the subject, "Dawn of the Health Age" (London: J. and A. Churchill, 1911, p. 162). He thinks that "if we were able to place 100,000 cases in sanatoria, choosing those most dangerous from the public health point of view, there is little doubt that the vast bulk of the dangerously infective cases would be removed from the midst of the community, and the effect of this would soon be obvious. From that time onward the recoveries and deaths in the sanatoria would rapidly make room for cases developing amongst the outside population, and soon the balance would set in the right direction."

Professor Moore estimates that at the outset a capital outlay of £10,000,000 (allowing an average of £100 for the provision of each bed) would be required to provide accommodation for these 100,000 patients. The annual cost of maintenance and treatment he estimates at six to seven million pounds (*i.e.*, £60 to £70 per head) at first, and this amount would rapidly diminish as the number of patients decreased.

For very advanced cases the colony scheme would offer no advantages. They might just as well be treated in the sanatoria or hospitals contemplated by Professor Moore. As the deaths from pulmonary consumption number about 50,000 per annum in this country there must be a sufficient number of consumptives already too advanced for work to occupy all the existing sanatorium accommodation, and I would urge upon public authorities the extreme importance of getting such cases out of crowded homes into suitable institutions, and suggest that the sanatoria now existing, or in course of erection, be used for advanced cases.

Those able to work could be treated in colonies at a much lower cost than £60 to £70 per head per annum, but on the other hand the capital expenditure involved by a colony scheme might be rather larger than that required for sanatorium accommodation. The capital expenditure would, however, be spread over a number of years and would be represented, to a large extent, by assets of permanent value. (After the eradication of tuberculosis the colony could be used in the treatment of other diseases or by the healthy population.) During the first few years when capital expenditure was heavy, maintenance charges would as yet be small, so that an expenditure of some seven millions a year might be made to cover both capital and maintenance charges until the system was in full working order, and as soon as the number of cases of tuberculosis became substantially reduced through the working of the system a progressive reduction of the cost would become possible. In order not to underestimate difficulties let us suppose that the cost will be even £10,000,000 a year for a few years. Compared with the sums which the Government has been accustomed to spend on public health this expenditure seems enormous, but when we compare it with the seventy-five millions per annum we are spending on the Army and Navy, or the 163 millions per annum which we spend on intoxicating liquors, and remember that tuberculosis is costing the country every year three or four times as many lives as we lost in the whole of the Boer war, and that a very conservative estimate puts the cost of consumption to the country in cash at £10,000,000 per annum, we shall see that after all if the remedy were really effective the price would be by no means extravagant. It comes to this, that for an annual expenditure equalling at first only one-seventh of the cost of our present Army and Navy, or one-sixteenth of our national drink bill, and gradually diminishing as time went on, we should be able to practically eradicate consumption from this country within the lifetime of a single generation. Some other facts ought to be borne in mind in this connection. The first is that the Government has made available under the Finance Act, 1911, the sum of £1,500,000 for the provision of, or making grants in aid of, sanatoria and other institutions. In the second place, the National Insurance Act provides sanatorium benefit, amounting to nearly £1,000,000 annually, for insured persons, and empowers Insurance Committees to include also the dependants of insured persons. The Act also provides that any estimated excess of expenditure over income may be met as to half by the County Council concerned, and as to the other half, from the Exchequer. In the third place, as announced by the Chancellor of the Exchequer on July 31, 1912, the "Government have decided to place at the disposal of the Local Government Boards (in Wales the Insurance Commissioners) of the three kingdoms annually a sum of money which will represent approximately half the total estimated cost of treating non-insured persons, as well as the dependants of insured persons."

The position is, therefore, that all tuberculous persons can be provided for, and any excess expenditure over and above that provided by the Insurance Act can be defrayed by the Exchequer and the local authorities. It is evident from this that most of the preliminary difficulties in the way of establishing such a scheme as I have outlined have already been cleared away, and that an excellent start has been made in making available the funds required for carrying it out. All that is required to bring it into being is the recognition by the authorities concerned that this scheme offers the most economical and effective method of applying sanatorium benefit, and of accomplishing the eradication of tuberculosis.

* The first article appeared on November 1.

It may be urged that this is an untried scheme and that public authorities would not be justified in spending public money on an experiment that might turn out to be a failure. Many successful experiments, however, have prepared the way for this scheme and established the principles on which it is founded. The Garden City at Letchworth is now an assured success, and the foresight of its founders has made it the healthiest town in England. In many sanatoria, such as Frindley and Benenden, graduated labour for patients has been in use for years, with excellent results. Even for epileptics, imbeciles, and feeble-minded industrial colonies, such as that of the Metropolitan Asylums Board at Darenth, with over 2,000 patients, have proved the most satisfactory form of institution. I quite agree, however, that just as the garden villages of Bournville and Port Sunlight preceded and made possible the Garden City, so in this matter it is desirable that a working model on a comparatively small scale be started first. This would afford valuable experience and demonstrate to all the soundness or otherwise of the plan.

The best means of providing such a working model and of taking the initiative in carrying the whole scheme through would be, I think, a voluntary National Association. Two existing associations—the National Association for the Prevention of Consumption and the Garden Cities and Town Planning Association—have hitherto been doing splendid work for the health of the nation independently of each other. If these two great Associations, with their powerful organisations and their long experience, could be induced to co-operate and form a joint committee to promote this scheme, I think success would be assured. The committee would collect money and draw up a definite scheme, and would hold public meetings and carry on an educational and propagandist campaign. The Welsh Memorial Association collected some £200,000. If England, with twenty times the population, could raise, say, £1,000,000, that would suffice for an experimental colony on an adequate scale. The committee would enter into arrangements with local authorities and with the Government, and with their co-operation would proceed to acquire a suitable site and start operations.

A harmonious combination of voluntary effort with that of the municipalities, the counties and the State would be, I think, the ideal way in which to carry the whole scheme through. Public authorities ought to be mainly responsible for the finance, but a noble field would be afforded for private beneficence which could help in countless ways to improve the amenities of the place and make life pleasant for the patients.

The scheme ought to be a national one. The essential feature is combination into sufficiently large units, and it will remain impossible if each local authority insists on running its own little show independent of the others. In the Welsh scheme all the County Councils and County Borough Councils have combined with the National Memorial Association for the purpose of discharging the whole of their duties with regard to tuberculosis. In England these duties, so far as imposed by the Insurance Act, are cast upon the County Councils, but the Act provides for a combination of two or more Councils. With regard to the provision of dispensaries and the necessary local staffs and institutions I see no advantage in greater centralisation than is already provided for, but for the purpose of establishing colonies for cases able to work, I think a national scheme in which all the counties would combine with a central association would be by far the best arrangement.

### The Argument for Segregation.

In this country about 50,000 people die of consumption each year (besides some 25,000 from other forms of tuberculosis), and it is pretty certain that there are at least 150,000 infectious cases at large in the community, and in more or less intimate contact with the rest of the population. One adult out of every seven, and in some trades, such as hotel servants and printers, one out of every three or four die of tuberculosis. Post-mortem examinations show that at one time or other the great majority of adults have been affected by the disease. It seems to me that this state of matters is a reproach to our civilisation and calls for a bold and comprehensive plan of action which shall aim at nothing less than the complete eradication of all sources of infection from the community. Tuberculosis is due to a parasite, the tubercle bacillus, and the proper way of dealing with parasitic diseases is to get rid of the parasites. I regret to say that doctors differ on this point. No less an authority than Professor Adami, of Montreal, is reported to have said in a recent lecture ("The Future of the Medical Profession," *Medical World*, Vol. I., No. 9, October 2, 1913): "I feel convinced that if we drive out such diseases as tuberculosis, typhoid and scarlet fever, we shall weaken the general bodily immunity. . . . It follows that I imagine no Utopia from which infection will be banished; only a state of generally improved conditions of existence in which the exanthemas and the severer infections will be much reduced in their incidence." To me this seems a monstrous doctrine. Would Professor Adami apply it to such comparatively harmless parasites as lice or fleas, tapeworms or ringworm—would he be satisfied if instead of being banished from his person and his home they were only "reduced in their incidence" as compared with places where they abound? If not, why tolerate such dangerous parasites as the tubercle bacillus? In this matter I think Moses showed himself a more advanced sanitarian than Adami, by sternly insisting on the removal of everything unclean "without the camp." Cleanliness is next to godliness, and infection is the most dangerous form of filth. We shall not be properly civilised until we have banished it in all its loathsome forms from our midst. Many forms of infection, plague, hydrophobia, typhus, smallpox, have already been rooted out of the country, and tuberculosis must be one of the next.

Many, however, who agree that this were a "consummation devoutly to be wished" are afraid that the task of eradication is an impossible one. The recent Departmental Committee on Tuberculosis declared in its final report: "It is idle to hope either that infection can be entirely eliminated, or that, apart from the discovery of some special process of immunisation, the resistant powers of human beings can be developed to such an extent that infection may be ignored." Here, again, doctors differ, for one of the members of the Departmental Committee says elsewhere ("The Conquest of Consumption," Latham and Garland. London: Fisher Unwin): "The disease does not exist in uncivilised countries until it is introduced, as it always is, in the train of civilising influences. . . . What civilisation produces civilisation should be able to stamp out. . . . We know how to prevent and how to cure consumption, and statesmen must now be made to realise that the eradication of the disease lies in their hands."

### Two Telling Facts.

In this connection two facts strike me as being of the greatest importance. The first is that referred to in the above quotation—viz., that among many races, such as the North-American Indians, the disease was unknown until the infection was introduced by white men. These people were nevertheless highly susceptible, for the disease once introduced spread like wildfire and proved very fatal.

The second fact is that, as demonstrated by Professor Bang, of Denmark, and his followers, large herds of cattle in which the disease is rife can be cleared of tuberculosis with great certainty and at little expense by the simple process of keeping the unaffected animals (including the new-born calves of tuberculous mothers) strictly separate from the diseased. Mr. J. Malcolm, veterinary superintendent of the Corporation of Birmingham, has been applying Bang's method with marked success in this country. He writes to me as follows: "I may say that from practical experience I know that we can clear herds by Bang's process of isolation provided two separate farms be employed, so that the infected animals are removed entirely from all possible contact with the free. Where the attempt is made to separate the infected from the free on the same farm the result is not entirely satisfactory. . . . We have now eleven herds, numbering 483 cows, free from tubercle, and we have another six herds being freed."

These two important facts seem to me to afford almost conclusive evidence that it is not "idle to hope," as the Departmental Committee declared, but quite reasonable to hope that infection can be entirely eliminated, and that in efficient segregation we have a reliable means of achieving that end.

The necessity for segregation in some cases is frankly recognised by the Departmental Committee, who state in their final report: "One of the principal sources of danger at the present time is the existence of a number of persons in the more acute and advanced stages of the disease living in the intimate contact with their families and neighbours which is necessitated by the ordinary conditions of their lives. The danger is frequently increased by the carelessness of the persons affected and of those living in contact with them. The Committee are of opinion that by means of treatment and education the risk of infection by such persons can be very largely diminished. But they do not think that treatment and education will be sufficient to eliminate all danger from this source. . . . The Committee desire, therefore, to recommend, as an effective means of preventing the spread of the disease, the compulsory isolation of certain cases which are in a state of high infectivity."

The measure of segregation advocated by the Committee is, however, a very limited one. They estimate that some 9,000 sanatorium beds should be provided, and that of hospital beds, in addition to Poor-Law beds, some 9,000 would be required for the purposes of observation, treatment, education, and isolation! This with 150,000 infectious cases in the community!

A very convincing study of the necessity for segregation is given by Professor Benjamin Moore in "The Dawn of the Health Age." He shows how widespread diseases have never been eradicated by "cures," no matter how potent these cures may be in individual cases. Treatment of those already affected is never sufficient. The removal of the cause—in the case of infectious diseases putting a stop to the spread of infection—is what is necessary. The administration of iron did not check the incidence of anæmia, nor the administration of quinine that of malaria, although both were potent remedies. Malaria and yellow fever, however, disappeared from many districts with dramatic completeness when the mosquitoes which carried the infection were exterminated. Professor Moore insists strongly on the need for drastic measures of segregation in dealing with tuberculosis, and I fully agree with his conclusion that in the general community, more especially in the crowded houses of the poor, it is quite impossible to enforce such precautions as to stop the spread of infection from "open" cases of consumption. So long as these infective cases remain at large, the cure of curable cases is like the operation of pumping a ship that has sprung a leak. Pump how you will, the water will keep flowing in until the leak is stopped. My objection to present measures against tuberculosis is that they are mainly devoted to pumping, while the leak is left gaping as wide as ever. Hundreds of fresh cases of infection occur daily. The consequence is that in spite of all our sanatoria and our sanitation, our doctoring and nursing, literally hundreds of cases die daily. Is this to be allowed to go on?

The objection most people have to a policy of segregation is almost entirely due to the assumption that it means imprisonment and an end to all joy and usefulness in life for the unfortunate victims. I maintain, on the contrary, that an intelligent segregation scheme would provide on the average better and happier conditions for the consumptive than he can find under present arrangements. The fact is that the general community is no place for the consumptive, as regards either his own interests or those of the public. As an American writer remarks: "The cure of consumption is not a drug, or an operation, or a magic method of any sort. It is a life that must be lived twenty-three hours and sixty minutes of the twenty-four. . . . At home the invalid has 'no one to play with.' He is 'odd man' at all the games of life. . . . He can compare himself only with his vigorous and healthy fellows, and his perpetual and insistent temptation is to try and live as they do, not as he must" (Woods Hutchinson: "Conquering Consumption." Constable, London, 1910).

In a well organised colony, on the other hand, he will find that "everybody is doing" what he has to do. His work will be skilfully adjusted to his powers. The constant temptation to outdo his strength will be removed. Instead of, it may be, a slum home and a badly ventilated factory or stuffy office, he will live and work in well-sunned, well-aired, and sanitary quarters. He will make friendships just as readily as he could outside.

It would be absurd to deny that segregation even in garden cities will entail hardships, through breaking up of domestic and business relations and separation from friends, but there will be ample compensations. Under our present *laisser faire* system, consumption is responsible for a vastly greater amount of those very same hardships without the compensations, and is annually carrying off from their families and friends tens of thousands of people just when their life ought to be at its prime—carrying them off not to colonies, where they can find fresh air, good food, suitable work, and the best chance of recovery, but through lingering, pauperising illness to the grave.

Like The Hospital's Special Commissioner the other day, I visited lately the wonderfully organised colony for imbeciles and feeble-minded at Darenth. I saw hundreds of these poor creatures at work, printing and bookbinding, making brushes and mats, boots and clothes, and I could hardly feel sorry for them, for they were clean and well cared for, happy as the day is long, living in wholesome surroundings and doing useful work. I thought of some village and slum idiots I have known, dirty and neglected, teased by the boys, "treated" to drink, and otherwise abused by men who ought to know better, and I thought what a strange obsession as to what segregation implies dominates the minds of even some members of Parliament!

Whether segregation is justifiable depends on how it is carried out. The trouble arises when it is attempted to segregate people in unsuitable and badly organised institutions. Let the institutions be on a large enough scale to afford liberty and variety; let site, buildings, and everything connected with them be intelligently designed for the purpose, and set kind-hearted and capable organisers to arrange and manage the whole, and segregation, whenever required in the interests of society, may be made a blessing instead of a blight to all concerned. Switzerland puts her tramps and wastrels into a colony and turns them into useful, self-supporting citizens. At New York Dr. Katherine Davis receives a constant stream of fallen women from the Law Courts—prostitutes, petty thieves, and other offenders—and after three years, or less, of colony training she sends them back to society for the most part physically sound, morally controlled and sufficiently skilled to be able to support themselves. These are examples of what intelligent segregation can do, even with the most unpromising material, and I feel sure that in the case of consumptives the results would be equally beneficent if the method were properly applied.

